# Disposable e-cigarette use and associated factors in US middle and high school students, 2021–2022

**DOI:** 10.18332/tid/189486

**Published:** 2024-06-26

**Authors:** Daniel T. H. Chen, Charis Girvalaki, Filippos T. Filippidis

**Affiliations:** 1 Nuffield Department of Primary Care Health Sciences, University of Oxford, Oxford, United Kingdom; 2 European Network for Smoking and Tobacco Prevention, Brussels, Belgium; 3 School of Public Health, Imperial College London, London, United Kingdom

**Keywords:** adolescent, e-cigarettes, nicotine addiction, tobacco control

## Abstract

**INTRODUCTION:**

Disposable e-cigarettes are the predominant type of vaping product used by adolescents and pose a significant public health concern. Identifying factors contributing to this growing trend is essential to curbing the vaping epidemic among youths. This study aims to investigate the growing prevalence and correlates of disposable e-cigarette use among US students.

**METHODS:**

Data from 48437 US middle and high school students from the 2021 and 2022 National Youth Tobacco Survey (NYTS) were analyzed using logistic and ordinal regression models to evaluate disposable e-cigarette use and frequency of use (low, medium, and high) with demographic and psychosocial factors. Weighted prevalence of current e-cigarette use with 95% CIs by device types in 2021 and 2022, were calculated. Odds ratios (ORs) of correlations of disposable e-cigarette use and frequency of use with demographic and psychosocial factors were analyzed.

**RESULTS:**

Disposable e-cigarette use increased from 3.9% (95% CI: 3.3–4.7) in 2021 to 5.1% (95% CI: 4.2–6.1) in 2022, and was associated with being female (OR=1.57; 95% CI: 1.29–1.91 vs male), high schoolers (OR=5.14; 95% CI: 3.96–6.67 vs middle schoolers), having low harm perceptions of e-cigarettes (OR=7.75; 95% CI: 5.58–10.75 vs lot of harm), and high exposure to marketing (OR=1.57; 95% CI: 1.05–2.35 vs low exposure). Identifying as LGBTQ (OR=1.41; 95% CI: 1.00–2.00 vs straight), having low academic performance (OR=2.16; 95% CI: 1.15–4.07, D vs A grades), and having psychological distress (OR=2.01; 95% CI: 1.64–2.47, severe vs none) were also linked to increased frequency of use.

**CONCLUSIONS:**

This study underscores increasing disposable e-cigarette use among US students, noting existing disparities. It identifies high-risk adolescent subgroups vulnerable to disposable e-cigarette use. These findings emphasize the urgency of targeted prevention and stricter regulations on disposable e-cigarettes to combat nicotine addiction among youths.

## INTRODUCTION

Disposable e-cigarettes have become increasingly popular among youth and have a leading role among vaping products^1^. Notably, these disposable devices have become the most commonly used type in the US and many European countries, contributing significantly to the rapid escalation of the vaping epidemic among adolescents^2,3^. Current use of disposable e-cigarettes among US high-school students soared from 0.7% in 2019 to 5.1% in 2020^1^, and from 0.1% to 10.7% among 18-year-olds in the UK between 2021 and 2022^1,4^.

Their attractive design, flavors, and ease of use have made these single-use devices especially attractive to youth, a trend intensified by targeted marketing^3^. The tobacco industry’s targeted campaigns have historically exacerbated health disparities, with e-cigarette use now disproportionately impacting racially or sexually minoritized youth and those with physical or mental health challenges^5,6^.

In 2020, the Food and Drug Administration (FDA) banned flavored e-cigarette sales^7^. However, unregulated disposable devices have grown even more popular among adolescents since then. Despite the FDA’s ban on sales to youths^8^, illegal use in this demographic remains a great public health concern^3,9,10^.

While there is a growing number of studies on e-cigarettes, a distinct gap exists in the focus on disposable e-cigarettes and the factors influencing their use. Using data from the 2021 and 2022 National Youth Tobacco Survey (NYTS), our study addresses a critical gap in existing evidence. We aim to investigate factors linked to disposable e-cigarette use and frequency of use among US students to improve understanding of the phenomenon and inform future decisions regarding relevant regulations.

## METHODS

We analyzed data from the 2021 and 2022 NYTS. The NYTS is a nationally representative, school-based, self-administered, electronically administered survey. The analysis pooled a total of 48437 students: 21804 from middle school (grades 6–8) and 26633 from high school (grades 9–12) across 2021 and 2022 of all students who undertook the survey during this time. Current use was defined as any use in the past 30 days.

Questions on demographic, psychosocial characteristics, e-cigarette device types, and use patterns are detailed in technical reports^11^. Study variables were selected based on existing literature on youth e-cigarette use, and CDC further guided their categorization reports^12^. Multivariable regression models were constructed using a step-wise method. We included variables identified from the literature as important predictors. The final specification of the models was informed by the Akaike Information Criterion (AIC), reinforcing the robustness of our approach.

The complex survey designs were accounted for in the statistical analyses using Stata 17^13^. We calculated weighted prevalence estimates for using various e-cigarette types across both surveys. Weighted logistic and ordinal regression models assessed how demographic and psychosocial factors were associated with disposable e-cigarette use among the total sample and frequency of use (categorized by 1–5, 6–19, and 20–30 days usage in the last 30 days)^12^ among current disposable e-cigarette users, respectively, adjusting for survey waves. The proportional odds assumption in the ordinal logistic models was assessed using appropriate statistical tests to ensure model validity. We set the level of statistical significance at 5%, and all tests were two-tailed. Results are presented as odds ratios (ORs) with 95% confidence intervals (CIs).

## RESULTS

The prevalence of current disposable e-cigarettes increased from 3.9% in 2021 to 5.1% in 2022 among US adolescents (Table 1); they were the most commonly used type in both years (accounting for more than half the students reporting e-cigarette use in both years).

**Table 1:** Weighted prevalence of e-cigarette use among middle and high school students by device types in the National Youth Tobacco Survey, United States, 2021 and 2022

	*2021 NYTS (N=20278)*				*2022 NYTS (N=28159)*			
	*Disposable*	*Refillable pods/ cartridges*	*Tanks/mod systems*	*Don’t know*	*Disposable*	*Refillable pods/ cartridges*	*Tanks/mod systems*	*Don’t know*
**Prevalence in NYTS sample,** n (%; 95% CI)	744 (3.9; 3.3–4.7)	422 (2.1; 1.7–2.6)	120 (0.7; 0.5–0.9)	118 (0.6; 0.5–0.8)	1443 (5.1; 4.2–6.1)	654 (2.3; 1.7–3.2)	206 (0.6; 0.5–0.8)	356 (1.2; 0.9–1.5)
	***2021 Current e-cig use (N=1404)***				***2022 Current e-cig use (N=2659)***			
	***Disposable***	***Refillable pods/ cartridges***	***Tanks/mod systems***	***Don’t know***	***Disposable***	***Refillable pods/ cartridges***	***Tanks/mod systems***	***Don’t know***
**Prevalence in current e-cig users,** %; 95% CI	53.7 48.8–58.6	28.7 25.0–32.6	9.0 6.8–11.8	8.6 6.7–11.0	55.3 49.4–61.0	25.2 19.7–31.5	6.7 5.3–8.4	12.8 10.2–16.1

The first row shows weighted prevalence for the total population (N=20278), and the second row shows prevalence within the current e-cigarette user population (N=1404) in each survey year. Current use defined as use of any e-cigarettes in the past 30 days. e-cig: e-cigarette.

**Figure 1:**
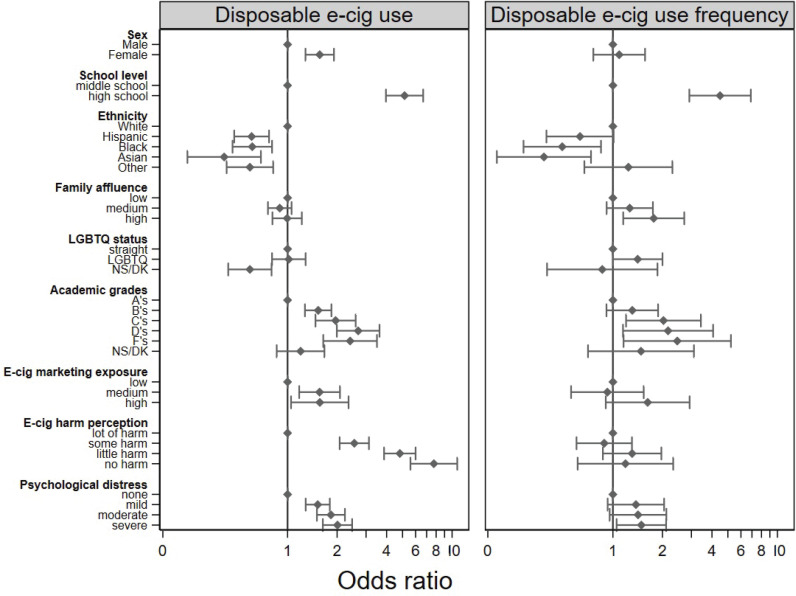
Estimation of association between individual-level factors and disposable e-cigarette use among middle and high school students (A) and frequency of use among current disposable e-cigarette users (B) in the National Youth Tobacco Survey, United States, 2021 and 2022

Analyses showed that female students had higher odds (OR=1.57; 95% CI: 1.29–1.91) of using disposable e-cigarettes than males (Figure 1). High schoolers were more likely (OR=5.14; 95% CI: 3.96–6.67) and more frequent users (OR=4.48; 95% CI: 2.91–6.89) than middle schoolers. Non-White ethnicities had lower likelihood and frequency of use than White students (OR<0.61 and OR<0.63, respectively). Affluent students were more likely to use them more frequently than their non-affluent peers (OR=1.77; 95% CI: 1.16–2.71). LGBTQ students had a higher frequency of use than their straight peers (OR=1.41; 95% CI: 1.00–2.00).

Students reporting lower grades had higher odds of using, and more frequent, disposable e-cigarettes than those who reported A grades (OR=2.69; 95% CI: 1.99–3.62 and OR=2.16; 95% CI: 1.15–4.07, respectively, for students reporting a D compared to an A grade). Increased exposure to e-cigarette marketing correlated with increased use (OR=1.57; 95% CI: 1.05–2.35 for students reporting high in comparison to low marketing exposure). Moreover, students perceiving e-cigarettes as having no harm were much more likely to use them (OR=7.75; 95% CI: 5.58–10.75) than those who acknowledged their risks. Additionally, students experiencing severe psychological distress were more likely to use disposable e-cigarettes than their less distressed peers (OR=2.01; 95% CI: 1.64–2.47).

## DISCUSSION

Our study underscores the high popularity of e-cigarettes among adolescents in the US and the disparities among youth e-cigarette use. We found that disposable e-cigarette use was associated with being female, in high school, and White. We also found increased disposable e-cigarette use among LGBT students, those reporting lower academic grades, and students with mental health concerns.

These highlight the disproportional impact of disposable e-cigarettes on the vulnerable subgroups of the youth population, especially sexual and gender minority adolescents and those experiencing mental health and academic stresses. This aligns with existing evidence on tobacco use and health inequalities^14^, suggesting that tobacco and nicotine products are more prevalent in disadvantaged groups who also bear greater physical and mental health burdens. Considering the popularity of disposable e-cigarettes, our findings are especially alarming and raise concerns regarding widening health and social inequalities, especially since youth exposure to nicotine and tobacco products is associated with the development of mental health disorders and respiratory issues^5^.

Our study also found that exposure to e-cigarette marketing and low perceptions of harm were linked to a higher likelihood of using disposable e-cigarettes. This confirms the significant impact of marketing and misconceptions of e-cigarette risks. The industry has been aggressively targeting adolescents through social media campaigns, promotional material, packaging, and product design. This strategy is particularly visible in disposable e-cigarettes, which may partly explain their commercial success. Past research has shown a positive association between increased advertising exposure and higher risks of e-cigarette use^15^, as well as how adolescents’ misconceptions about e-cigarettes’ health risks contribute to their widespread use^16^. Disposable e-cigarettes are not an exception, and their use continues to be driven by such factors.

### Strengths and limitations

We analyzed nationally representative data from a big sample of US adolescents and were able to explore a range of sociodemographic and psychosocial characteristics. However, the cross-sectional design prevents causal inferences. We relied on self-reported data for both ever use and frequency of use, which may be subject to recall bias. Additionally, there may be residual confounding despite adjustments for various factors. Lastly, the findings may have limited generalisability to youths in other countries with different sociodemographic and regulatory contexts. Further research could shed light on the patterns of use, preferred flavors, and sources of disposable e-cigarettes among young users.

## CONCLUSIONS

Our findings highlight not only the alarmingly high prevalence of disposable e-cigarettes in the US and driving a resurgence of e-cigarette use but also the existing disparities and, hence, the urgent need for targeted interventions for minority adolescents at higher risk of use. As the vaping landscape continues to evolve, stronger regulations on sales and marketing around e-cigarette products, particularly disposable devices, could focus on prevention among at-risk groups and educational campaigns with mental health support to mitigate the harmful impact of disposable e-cigarettes on youth.

## Data Availability

The data supporting this research are available from the authors on reasonable request.
